# Conservative management of odontogenic keratocyst with long-term 5-year follow-up: Case report and literature review

**DOI:** 10.1016/j.ijscr.2019.11.023

**Published:** 2019-11-20

**Authors:** Kleber A. Vallejo-Rosero, Gisela Vianna Camolesi, Pedro Luiz Duarte de Sá, Wilber E. Bernaola-Paredes

**Affiliations:** a Department of Oral and Maxillofacial Surgery, School of Dentistry, Universidad Central del Ecuador, Quito, Ecuador; b Department of Oral Diagnosis, School of Dentistry, University of Sao Paulo, Sao Paulo, Brazil; c Department of Stomatology, A.C. Camargo Cancer Center, Sao Paulo, Brazil

**Keywords:** Odontogenic keratocyst, Surgical treatment, Therapeutic approaches, Recurrence, Odontogenic cyst

## Abstract

**Introduction:**

Odontogenic Keratocyst (OKC) is a benign lesion recently considered an odontogenic cyst in the present World Health Organization (WHO) classification of Head and Neck tumors (2017) mainly based on its clinical and histopathological features. Several studies, systematic reviews and meta-analyses have shown a multimodal approach to reducing the high recurrence rates inherently exhibited by this pathological condition after long-term periods of follow-up.

**Presentation of case:**

The patient, a 67-years-old woman, was submitted to conservative surgical management of an odontogenic keratocyst. Initially this consisted of decompression and subsequent enucleation, with a long-term period of follow-up to ensure that there was no recurrence.

**Discussion:**

Conservative surgical management is the first choice and might be considered the gold standard, but combined therapy such as marsupialization, application of Carnoy’s solution, enucleation of the remnant lesion and an extensive follow-up period would considerably reduce recurrence rates.

**Conclusion:**

Therefore, an appropriate long-term follow-up must be done after the treatment performed in order to ensure clinical success, which in this case was described as an absence of signs of recurrent disease.

## Highlights

• The World Health Organization, in the last classification of the Head and Neck tumours defined Odontogenic Keratocyst as a cyst instead of a tumor.
• There are plenty of approaches in order to reduce the high recurrence of this lesion consisted in surgical, non-surgical and combined treatment.
• Surgical treatment is considered, for several years as the gold standard treatment, but currently a combined therapy has become as a first choice.

## 1 Introduction

Odontogenic keratocysts (OKC), recently considered in the World Health Organization (WHO) classification of 2017 was now re-classified as a cystic lesion of benign nature, with a prevalence of 10 % [1], among other lesions of the maxillofacial region such as hamartomas, bone cysts odontogenic and neoplasms. Another author [2] stated that the OKC has an embryonic origin from the cellular remnants of the dental lamina.

The first case reported and described in the literature was by PHILLIPSEN et al. [3]; who named it an Odontogenic Keratocyst. In 2005, after the publication of several series of cases reporting specific characteristics such as the aggressive behavior of the lesion, its slow growth and high recurrence rate, the World Health Organization (WHO) included it in the list of benign odontogenic tumors and it was denominated a keratocyst odontogenic tumor (KOT); However, after 12 years, this denomination was reconsidered and it was reclassified as an odontogenic cystic lesion, by the WHO in 2017.

PAYNE et al. [4], reported no differences with reference to predilection for gender in the clinical diagnosis of the OKC; whereas, BRANNON et al. [1] reported a certain predilection for the male gender. These lesions could be diagnosed at any age stage; however, 60 % of cases occurred in individuals between 10 and 40 years of age [5]. On the other hand, regarding the topographic sites for the occurrence of OKC, the majority of studies showed that they were diagnosed mainly in the body, ascending branch and mandibular angle [6].

In the vast majority of OKC cases, an absence of any type of symptomatology was found, because of it is usually discovered during routine imaging exams (Orthopantomography). In addition, the growth rate of this pathological condition is slow, and occurs in the anteroposterior direction, covering the medullary spaces of the bone. Consequently, in the majority of cases, in the initial stages, it is not possible to show it through the clinical finding of bone cortical swelling.

In imaginological assessment, the OKC is characterized by the presence of a single or multillocular radiolucent area, with well-defined edges and regular margins. The larger lesions are often multillocular. In many cases, an unerupted tooth is associated with the cystic lesion [4], which may raise doubts regarding the definitive diagnosis. It is possible to make the differential diagnosis with lesions such as the dentigerous cyst, ameloblastoma, calcifying odontogenic cyst, adenomatoid odontogenic tumor (AOT) and ameloblastic fibroma [7].

Relative to the pathological analysis, the OKC has a thin and friable capsule, with approximately eight to ten layers of epithelial cells. The intraluminal space visualized is composed of a cheesy material that shows evidence of the presence of keratin in its interior [2].

In order to confirm the diagnosis of OKC, it is necessary to perform a complete intra and extra-oral clinical evaluation, thorough radiographic analysis and particularly the pathological examination to establish an accurate final diagnosis.

Different treatment modalities have been described, ranging from conservative techniques to radical surgeries. Concerning the conservative approach, marsupialization and simple enucleation have shown high recurrence rates. For this reason, the combination of therapies has been selected in recent approaches reported in the literature, such as peripheral ostectomy performed with drill bits, cryotherapy and the use of chemical chelation by applying Carnoy’s Solution [8]. On the other hand, techniques that are more radical are indicated in the treatment of lesions that involve adjacent soft tissues and in which there is evidence of cortical bone rupture. In these cases, the use of bone resection surgery with a free margin would be justified due to the high risk of recurrence and pathological fracture.

The OKC has a relatively high recurrence rate, around 7–28 % during the first 5 years after treatment. This variation occurs due to some of the diagnostic criteria and as a consequence of the treatment, taking into consideration appropriate use of the different techniques described above, location and extent of the lesion [9]. Therefore, a long-term follow-up is recommended before and after the surgical treatment performed. Meanwhile, the prognosis is favorable.

The aim of this case report was to describe the results obtained with a conservative surgical approach in a patient diagnosed with odontogenic keratocyst and after a 5-year follow-up, in contrast with the results reported in the contemporary literature. This study was reported in line with the SCARE criteria [10].

## 2 Case report

**Fig. 1:**
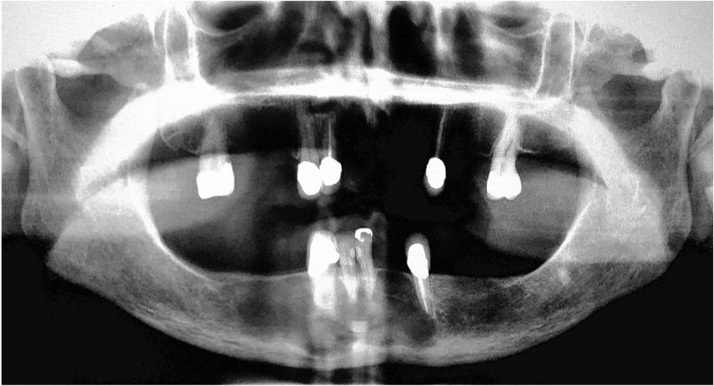
Orthopantomography with evidence of radiolucency in the submental and mental region of mandible bone.

The patient, a 67-years-old woman, who came to the first clinical consultation with a history of hypothyroidism and allergy to aspirin and penicillin, with painful symptoms in the submental and mental right side of mandibular bone region, with a duration of 3 months. The patient was attended by professionals of the Department of Dentistry, who proposed a procedure of surgical extraction of the mandibular teeth involved in the anterior section. Orthopantomography was performed, in which a large radiolucent area was observed in the chin region, so for this reason, she was referred to the Oral and Maxillofacial Surgery Department (Fig. 1).

**Fig. 2:**
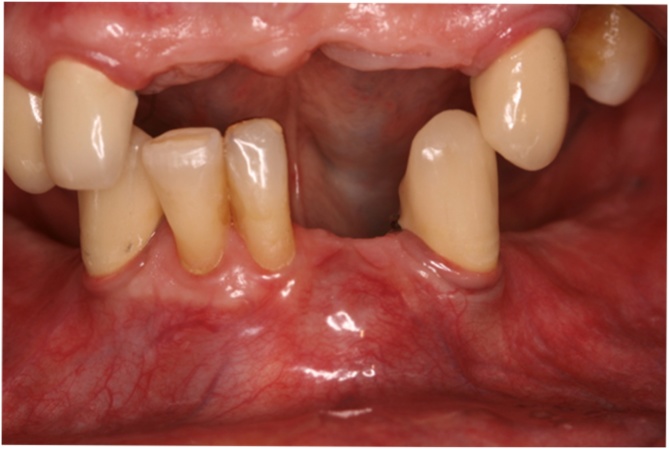
Cortical vestibular bone expansion of the anterior region in mandible bone at intraoral clinical examination.

After medical and dental details described above, an intraoral clinical assessment of the area was made, in which an increase in volume of the vestibular bone table//plate?/ was observed, tooth mobility between grade 1–2 found on inspection of 4.1–4.2. On the other hand, teeth 3.3 and 4.3 showed no pathological mobility. In addition, the surrounding mucosa was erythematous, especially in the region of teeth 3.1 and 3.3, in which the tissue was fluctuating due to possible perforation of the cortical vestibular bone as seen in Fig. 2.

Computed tomography (CT) assessment showed the presence of a delimited, unilocular, hypodense zone along its entire perimeter, limited by a thin bone cortex on the one hand, from the mesial-apical region of teeth 33–46, below the mandibular basilar edge. There was a short conservation of the basilar border, especially in the midline with greater resorption on both sides, so that greater involvement was shown in the upper part of the alveolar ridge, involving the teeth present, in the mesial and apical regions of tooth 33, and teeth 41, 42, 43.

**Fig. 3:**
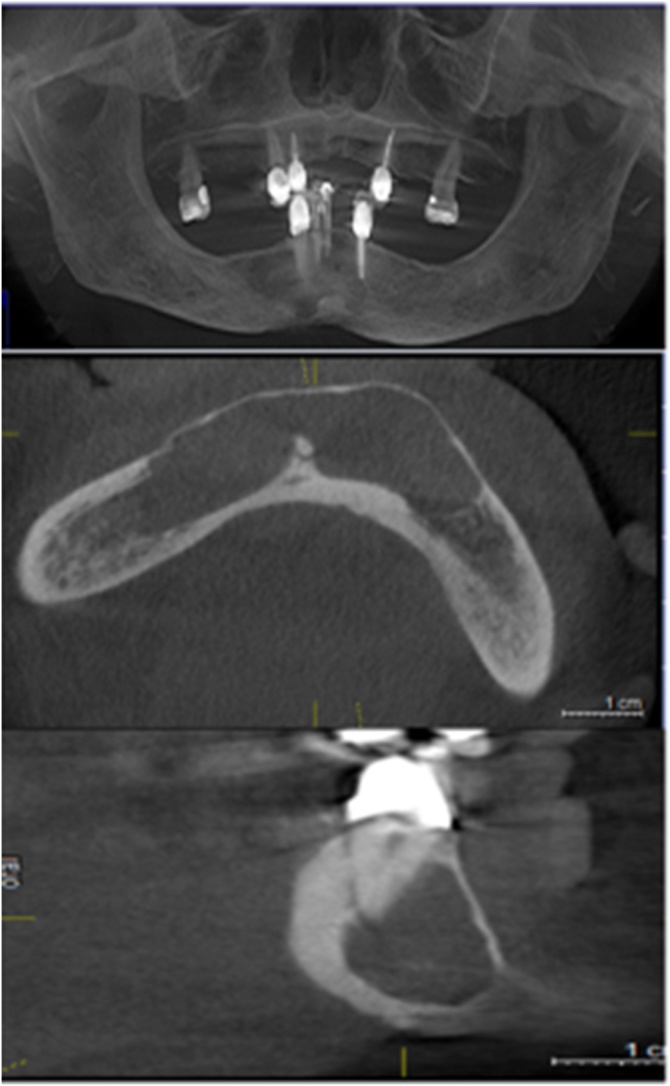
Orthopantomography and CT image showing frontal and sagittal slices of the tumor region.

In the region itself of these teeth, an accentuated loss of bone support was found, at the level of the middle and apical third, only the cervical third of the roots was preserved. On the other hand, a smaller amount of resorption of the lingual bone and greater resorption of the vestibular cortical bone were observed throughout the extent of the lesion, measuring a thickness of only 1–1.5 mm, as shown in Fig. 3.

**Fig. 4:**
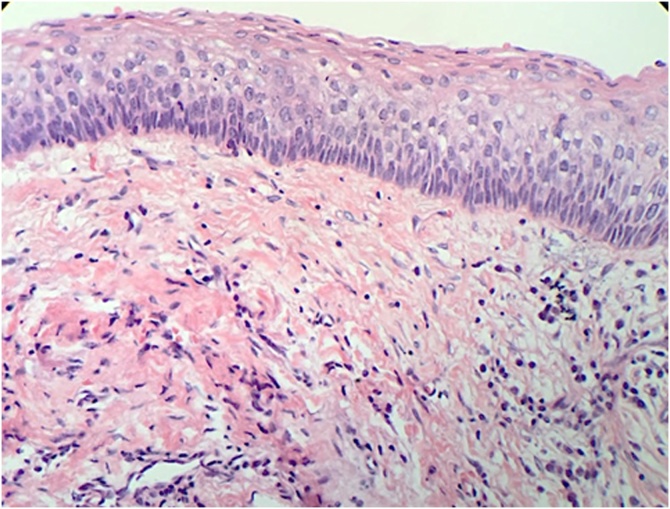
Histopathological assessment with Hematoxylin and Eosin staining (H&E), confirmed our initial diagnostic.

As the clinical and radiographic examination of the lesion was performed and having made an initial diagnosis compatible with odontogenic keratocyst, it was decided to perform a fine needle aspiration puncture of the content for subsequent pathological analysis. Thereby, a milky-yellow liquid content of approximately 10cc was obtained. In addition, the lesion showed a thick cystic capsule of intense red wine color, which showed some degree of resistance to surgical excision. Both surgical pieces were analyzed histopathologically confirming the initial diagnosis of moderate-grade unilocular Parakeratinized Odontogenic Keratocyst (former classification of odontogenic keratocyst that was reclassified as a keratocyst odontogenic tumor) as shown in Fig. 4.

After 30 days of initial decompression of the lesion, surgical resection of the lesion was performed under general anesthesia. A supracrestal incision was made in the mandibular residual edentulous alveolar ridge, covering the teeth present and extending bilaterally to the first molar regions. After this, the mucoperiosteal flap was lifted, exposing the vestibular cortical bone to obtain a complete and improved direct view of the lesion.

**Fig. 5:**
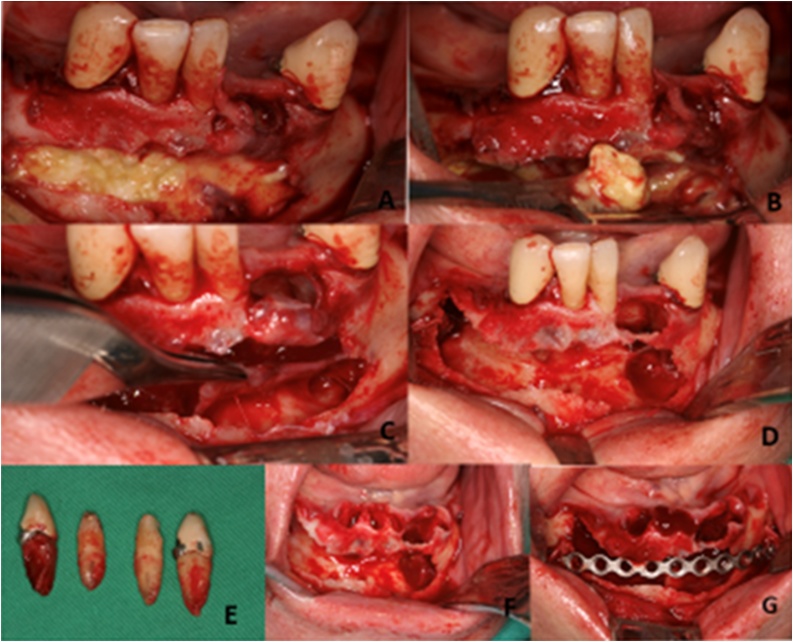
Sequence of conservative surgical management of keratocyst odontogenic lesion and reconstruction with plates and screws to diminish the risk of pathological fracture of the mandible bone.

Subsequently, a peripheral osteotomy was performed to enable direct visualization of the odontogenic keratocyst, using tungsten carbide surgical drills and a gouge clamp. In addition to the surgical procedure of excision of the lesion, enucleation and vigorous curettage were performed. The remaining teeth were removed and Carnoy’s solution was applied as an agent for chelating the remaining bone tissue, embedded in a gauze for 3 min. Finally, a reconstruction plate of the unlock 2.0 system was positioned to cover the middle portion of the mandibular body and extending from the region of the right first right molar to the left first molar. This was stabilized with 7 mm long mono-cortical screws to prevent a possible inter- or postsurgical mandibular fracture. The anatomical layers were repositioned using simple stitches with Vicryl 4.0 (Fig. 5).

**Fig. 6:**
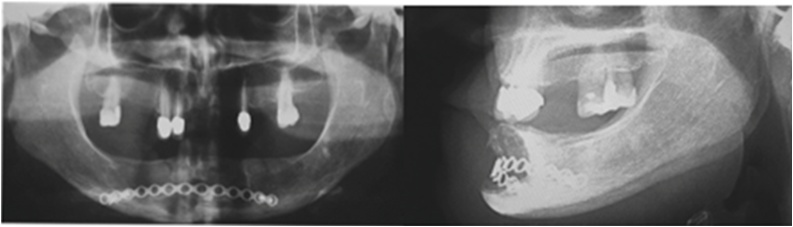
Orthopantomography 3 months after surgical procedure and it was visualized an establish process of healing.

Furthermore, post-surgical control was performed for the removal of the stitches, observing adequate healing of the postoperative wound, without signs of dehiscence and surgical bed infection. In addition, plans were made for follow-up consultations a month later, at three months, and at one year, with control panoramic radiography, in which satisfactory functional results were observed and without signs of recurrence, as shown in Fig. 6.

**Fig. 7:**
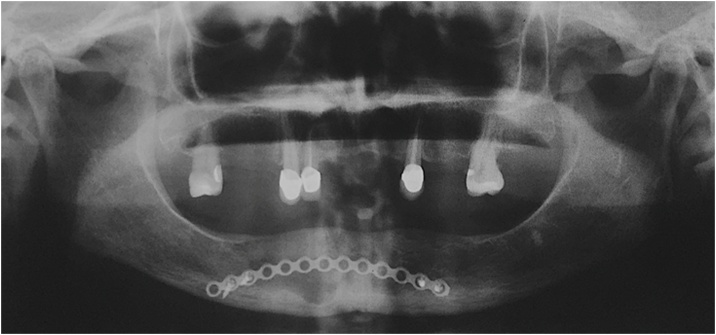
Follow-up of 6 months after surgical procedure without evidence of lesion recurrence.

Clinical and radiographic control was performed after 6 months of follow-up, and a favorable healing process was observed without signs of recurrence of the lesion, as may be seen in Fig. 7.

**Fig. 8:**
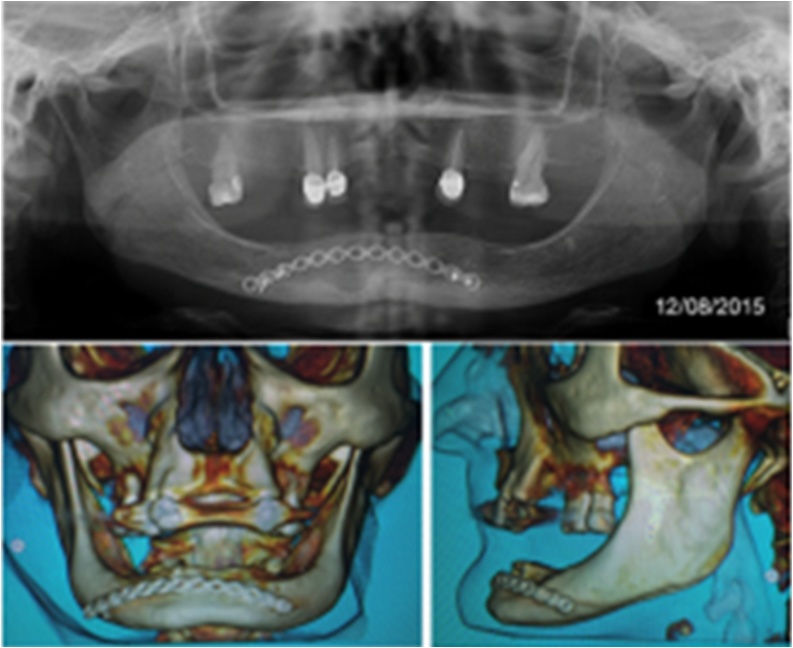
Follow-up of two years and it was observed a normal healing process and bone neoformation surrounding osteosynthesis material.

After two years of follow-up; a satisfactory bone remodeling of the region that underwent surgery was found, showing bone characteristics similar to those of the surrounding normal bone tissue. Correct positioning of the osteosynthesis material was also verified, with the absence of resorption areas around it (Fig. 8).

**Fig. 9:**
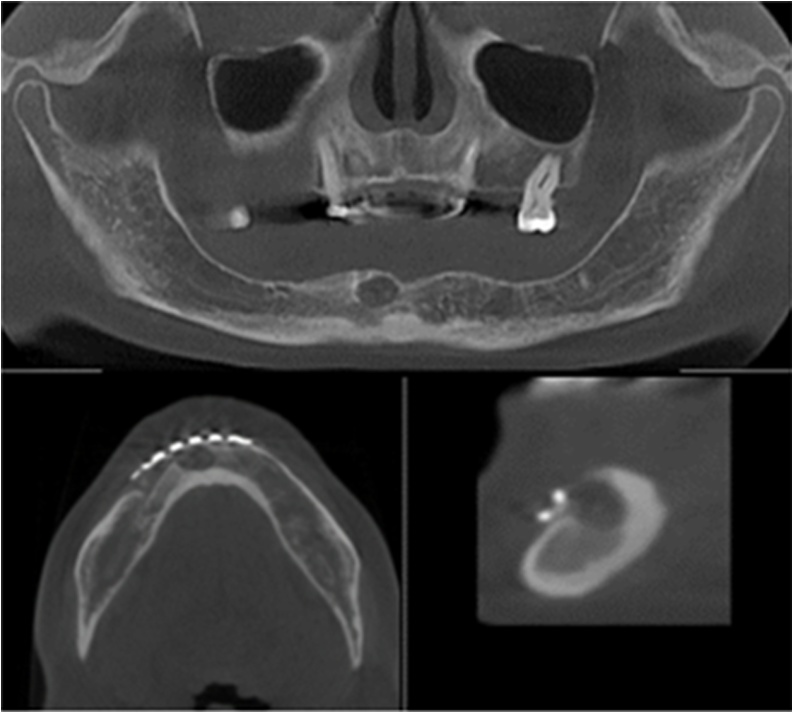
CT images assessment showed areas radiolucent surrounding the region submitted to surgical procedure.

In an accurate CT assessment after the two year follow-up, isolated radiolucent areas, approximately 1 cm in diameter were identified in the region of teeth 4.1 and 4.2, which was compatible with recurrence of lesion, and therefore it was decided to undertake a second surgical procedure in order to obtain the diagnosis of recurrence. The osteosynthesis material was removed, as shown in Fig. 9.

**Fig. 10:**
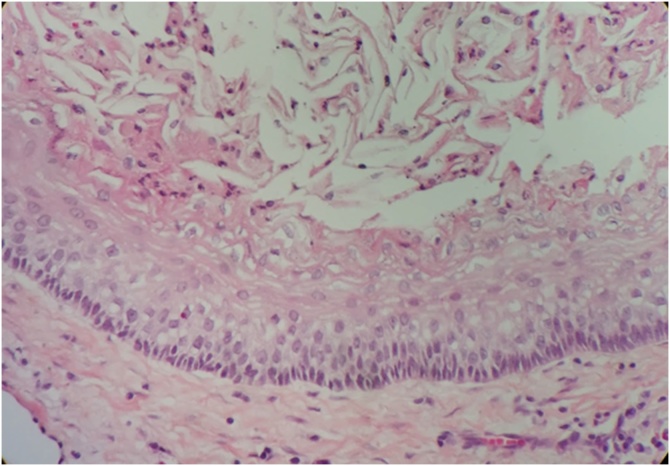
Recurrence lesion of parakeratinized odontogenic keratocyst in mandible bone confirmed by histopathological assessment.

**Fig. 11:**
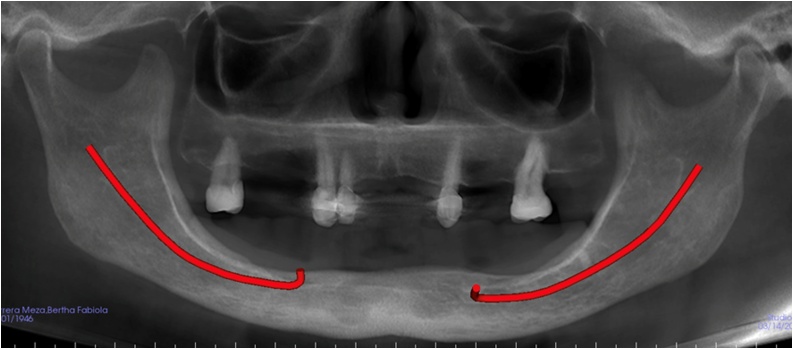
Areas of new bone formation after surgical conservative approach of local recurrence with 5 years follow-up.

Therefore, the histopathological assessment performed the second time confirmed a recurrence of the initial odontogenic keratocyst - parakeratotic subtype as shown in Fig. 10. A new conservative surgical approach was performed to remove the recurrent lesion and patient continues with control follow-up to ensure absence of new local recurrence. In the last Orthopantomography performed, a local area with new bone formation was noted instead of a local lesion appearance as may be visualized in Fig. 11.

## 3 Discussion

The spectrum of treatment modalities has been established in the literature since 2005 as conservative and non-conservative approaches to Odontogenic Keratocysts (OKC). Conservative methods described as simple enucleation, decompression or marsupialization and other techniques that are aggressive or non-conservative approaches such as cryosurgery or chemical destruction and radical surgery with bone resection [6,11]. However, there is no consensus or adequate evidence for determining which is the most appropriate or appropriate technique for treating OKCs. The use of complementary techniques such as Carnoy’s solution and liquid nitrogen cryotherapy may cause unwanted side effects [6].

A recent systematic review and meta-analysis based on the rates of KOC recurrence in patients submitted to conservative surgical treatments showed considerable results, which may vary significantly depending on the type of approach applied. Moreover, in a high percentage of cases reviewed in this study, almost 19.8 % developed a local recurrence of the initial pathological condition. On the other hand, lower recurrence rates were found in cases in which more aggressive treatments were applied, such as bone resection (0 %), 7.8 % for cases treated by enucleation and Carnoy’s solution, and 11.5 % for cases treated by enucleation and liquid nitrogen [12]. Nevertheless, previous studies have pointed out the importance of a considerable length of follow-up of these lesions, because some cases showed no recurrence in a short-term follow-up, and this would be considered a weak point in most of the published articles [6].

In the present case report, decompression and enucleation alone were performed as the first-choice treatment, and the use of screw-retained plate to avoid pathological fracture in the residual bone. In the contemporary literature, it was recommended and established that more aggressive treatment have many disadvantages when compared with the clinical results of the conservative approach [12], because the former produced significant morbidity such as facial deformities, loss of bone continuity (maxillary/mandible) reported in literature [[12], [13], [14], [15]].

Carnoy’s solution, initially used as a slide fixator in laboratory pathology [16], is applied in the bone cavity for the purpose of eliminating the tumour tissue remnants by promoting a superficial chemical necrosis of up to 1.5 mm^2^. In a previous study, which evaluated the clinical results observed with the use of Carnoy’s solution, suture dehiscence and postoperative infection occurred, in addition to nerve injuries causing temporary toxicity paresthesia due to the undesirable effect caused by Carnoy’s solution applied directly on the inferior alveolar nerve, determined by exposure of the nerve in these reported cases [17]. Moreover, other studies argued about a time of critical exposure [[18], [19], [20]]; however, studies that supported the use of Carnoy’s solution denied that it had a prolonged toxic effect on nerve tissues [21].

There are others effects mentioned in the contemporary literature [20,21] such as irreversible damage to the superficial and devitalized bony margin [11,21]; but in our case reported we used a Carnoy’s solution directly on the remnant alveolar ridge after lesion removal and no signs of toxicity were found in both the first a second surgery performed.

Moreover, enucleation alone followed by peripheral ostectomy and application of Carnoy’s solution was chosen in order to promote the complete removal of cell elements associated with OKC, with the aim of minimizing the potential for recurrence and postoperative complications [[20], [21], [22]].

Thus, clinical aspects, tumor location and particularly the tomographic examination of the lesion justified the approach adopted, since there was no bone fenestration and lesion communication with the surrounding soft tissues. The integrity of the surgical space was observed and the surgical planning ruled out the necessity for adjacent soft tissue excision. Moreover, the macroscopic aspects of the lesion with integrity of the fibrous capsule during curettage reaffirmed the choice of peripheral ostectomy, as was performed in the initial surgical management [22].

Therefore, our case report supported the idea of conservative management of OKC, using combined therapy with an initial decompression and enucleation as described above. However, the recurrence rates continue to remain high, therefore extended long-term follow-up, with an established periodicity of months or years is necessary, in order to avoid further surgical procedures that diminish the quality of life, as well as the functional and esthetic changes that occur in patients have undergone this type of treatment.

## 4 Conclusion

Conservative surgical management of Odontogenic Keratocyst (OKC) with combined therapy using multimodal therapeutic approaches was shown as the first choice for treating this pathological condition, which showed a recurrence two years after the first surgical procedure. Therefore, an appropriate long-term follow-up must be done after the treatment performed in order to ensure clinical success described as an absence of signs of recurrent disease. We encourage further prospective studies to be performed in order to assess the multimodal approach and to elucidate the role played by each approach in reducing the disease recurrence rates.

Written informed consent was obtained from the patient for publication of this case report and accompanying images. A copy of the written consent is available for review by the Editor-in-Chief of this journal on request.

## Conflicts of interest

There are not conflicts of interest for this article to declare.

## Funding

This work was supported by the National Council for Scientific and Technological Development of Brazil (CNPq) (870091/2001-8).

## Ethical approval

This report was exempt for ethical approval.

## Consent

Written informed consent was obtained from the patient for publication of this case report and accompanying images. A copy of the written consent is available for review by the Editor-in-Chief of this journal on request.

## Author contribution

All authors have contributed in the each step for writing this paper, participating in the whole process to retrieve medical information, review of the literature, and writing of each issue included.

Wilber Edison Bernaola-Paredes has written and selected the topics for structuring this case report. Moreover, he did and worked in the Introduction and Discussion issues. On the other hand, Gisela Camolesi has done the literature review in order to support our discussion and introduction. Kleber Vallejo-Rosero did the case report (presentation and details about it) and took the all pictures during the surgery performed and follow-up in the clinical attendance. Kleber Vallejo was the first oral and maxillofacial surgeon who performed odontogenic keratocyst (OKC) surgery management, Wilber and Gisela participated as members of this team.

## Registration of research studies

It is a case report, observational, descriptive and retrospective study in which we have retrieved medical information from patient’s records.

## Guarantor

Wilber Edison Bernaola-Paredes/Kleber Vallejo-Rosero.

## References

[1] Brannon R.B. (1976). The odontogenic Keratocyst: a clinicopathologic study of 312 cases. Part I. Clinical features. Oral Surg. Oral Med. Oral Pathol..

[2] Neville B.W., Damm D.D., Allen C.M., Bouquot J.E. (2016). Oral and Maxillofacial Pathology.

[3] Philipsen H.P. (1956). Om keratocyster (kolesteatom) I kaeberne. Tandlaegebladet.

[4] Payne T.F. (1972). An analysis of the clinical and histopathologic parameters of the odontogenic keratocyst. Oral Surg. Oral Med. Oral Pathol. Oral Radiol. Oral Endodontol..

[5] Meara J.G., Li K.K., Shah S.S. (1996). Odontogenic keratocysts in the pediatric population. Arch. Otolaryngol. Head Neck Surg..

[6] Kaczmarzyk T., Mojsa I., Stypulkowska J. (2012). A systematic review of the recurrence rate for keratocystic odontogenic tumour in relation to treatment modalities. Int. J. Oral Maxillofac. Surg..

[7] Bsoul S.A., Paquette M., Terezhalmy G.T., Moore W.S. (2002). Odontogenic keratocyst. Quintessence Int..

[8] Fonseca E.V., Franzi S.A., Marcucci M., De Almeida R.C. (2010). Clinical, histopathological and treatment factor of the odontogenic keratocyst. Braz. J. Head Neck Surg..

[9] Antonoglou G.N., Sándor G.K., Koidou V.P., Papageorgiou S.N. (2014). Non-syndromic and syndromic keratocystic odontogenic tumors: systematic review and meta-analysis of recurrences. J. Craniomaxillofac. Surg..

[10] Agha R.A., Borrelli M.R., Farwana R., Koshy K., Fowler A., Orgill D.P., For the SCARE Group (2018). The SCARE 2018 statement: updating consensus Surgical CAse REport (SCARE) guidelines. Int. J. Surg..

[11] Morgan T.A., Burton C.C., Qian F. (2005). A retrospective review of treatment of the odontogenic keratocyst. J. Oral Maxillofac. Surg..

[12] Santos de Castro M., Caixeta C.A., De Carli M.L., Júnior N.R., Miyazawa M., Pereira A.C. (2018). Conservative surgical treatments for nonsyndromic odontogenic keratocyst: a systematic review and meta-analysis. Clin. Oral Investig..

[13] Tolstunov L., Treasure T. (2008). Surgical treatment algorithm for odontogenic keratocyst: combined treatment of odontogenic keratocyst and mandibular defect with marsupialization, enucleation, iliac crest bone graft, and dental implants. J. Oral Maxillofac. Surg..

[14] Da Silva Y.S., Stoelinga P.J.W., Naclério-Homem M.G. (2019). Recurrence of nonsyndromic odontogenic keratocyst after marsupialization and delayed enucleation vs. enucleation alone: a systematic review and meta-analysis. Oral Maxillofac. Surg..

[15] Da Silva Y.S., Stoelinga P.J.W., Naclério-Homem M.G. (2019). The presentation of odontogenic keratocyst in the jaws with na emphasis on the tooth-bearing área: a systematic review and meta-analysis. Oral Maxillofac. Surg..

[16] Pogrel M.A. (2013). The keratocystic odontogenic tumor. Oral Maxillofac. Surg. Clin..

[17] Ribeiro Júnior O. (2012). Study of Surgical Treatment of Keratocystic Odontogenic Tumors Associated or Not to the Nevoid Basal Cell Carcinoma Syndrome and Analysis of Recurrence-free Period.

[18] Chapelle K.A.O.M., Stoelinga P.J.W., De Wilde P.C.M., Brouns J.J.A., Voorsmit R.A.C.A. (2004). Rational approach to diagnosis and treatment of ameloblastomas and odontogenic keratocysts. Br. J. Oral Maxillofac. Surg..

[19] Frerich B., Cornelius C.P., Wietholter H. (1994). Critical time of exposure of the rabbit inferior alveolar nerve to Carnoy’s solution. J. Oral Maxillofac. Surg..

[20] Belenguer A.D., Sánchez-Torres A., Gay-Escoda C. (2016). Role of carnoys solution in the treatment of keratocystic odontogenic tumor: a systematic review. Med. Oral Patol. Oral Cir. Bucal.

[21] Stoelinga P.J.W. (2001). Long-term follow-up on keratocysts treated according to a defined protocol. Int. J. Oral Maxillofac. Surg..

[22] Johnson N.R., Batstone M.D., Savage N.W. (2013). Management and recurrence of keratocystic odontogenic tumor: a systematic review. Oral Maxillofac. Surg..

